# Large language models and multimodal foundation models for precision oncology

**DOI:** 10.1038/s41698-024-00573-2

**Published:** 2024-03-22

**Authors:** Daniel Truhn, Jan-Niklas Eckardt, Dyke Ferber, Jakob Nikolas Kather

**Affiliations:** 1 Department of Diagnostic and Interventional Radiology, University Hospital Aachen, Aachen, Germany; 2 Department of Internal Medicine I, University Hospital Carl Gustav Carus, Technical University Dresden, Dresden, Germany; 3 Else Kroener Fresenius Center for Digital Health, Technical University Dresden, Dresden, Germany; 4 National Center for Tumor Diseases (NCT), Heidelberg University Hospital, Heidelberg, Germany; 5 Department of Medical Oncology, Heidelberg University Hospital, Heidelberg, Germany

**Keywords:** Cancer, Oncology, Mathematics and computing

## Abstract

The technological progress in artificial intelligence (AI) has massively accelerated since 2022, with far-reaching implications for oncology and cancer research. Large language models (LLMs) now perform at human-level competency in text processing. Notably, both text and image processing networks are increasingly based on transformer neural networks. This convergence enables the development of multimodal AI models that take diverse types of data as an input simultaneously, marking a qualitative shift from specialized niche models which were prevalent in the 2010s. This editorial summarizes these developments, which are expected to impact precision oncology in the coming years.

The volume of patient-specific data in oncology is rapidly expanding. This is due to the widespread introduction of electronic health records (EHRs), advances in medical imaging, and the integration of large-scale genomic analyses into clinical routine. Effective use of this large amount of data is important for ensuring the optimal treatment for cancer patients. Artificial intelligence (AI) and machine learning (ML) have shown promise in helping healthcare professionals to process such data.

AI’s application in domains like oncology has experienced periodic surges of activity. Prior to 2012, AI technologies played a marginal role in oncology research. Computer-based data analysis studies mostly relied on classical ML algorithms with a modest model complexity. 2012 marked a turning point in computer-based data analysis: image processing, a notoriously difficult task, was suddenly made much easier with the advent of convolutional neural networks (CNNs). This technological shift was subsequently integrated into medical research^1^, most notably evidenced by a 2017 publication demonstrating neural network performance on par with human experts across large image datasets^2^. In parallel, hardware improvements have gradually lowered the computational barriers for training and deploying resource-intensive models, thus broadening the user base capable of developing and refining AI algorithms^3^.

Between 2012 and 2022, neural networks were applied in numerous studies that primarily focused on the analysis of oncological imaging or text^1^. Regulatory bodies in the United States and the European Union approved a number of specialized AI-based tools for cancer, particularly in radiological and pathological image analysis. However, high-profile and ambitious initiatives, such as IBM Watson, did not achieve their projected outcomes^4^. This decade can be viewed as a stabilization phase for AI applications in oncology, marked by incremental improvements and specialized uses, predominantly in image analysis^5^. This landscape changed in 2022 and 2023 with the advent of two key innovations: large language models (LLMs) and multimodal AI models.

LLMs are deep learning models that serve the purpose of both processing and generating primarily text-based data^6^. The training data for these models is a large and diverse amount of text, usually sourced from the internet and commercial data providers, and can include diverse types of medical data^7^. While potentially this purpose can also be fulfilled by various model architectures, the most successful models have recently relied on transformer-based architectures pertaining to their attention mechanisms^8^. Their training process is autoregressive, which means that the model is trained to predict subsequent tokens in a sequence (similar to words in a sentence). Notably, model performance scales with size, i.e. number of parameters, and larger models show emergent behavior^9^: They acquire an understanding of concepts underlying the training data without having been explicitly trained on this. When LLMs are applied on new tasks without explicit training, this is referred to as a “zero-shot” application. The LLM Generative Pre-trained Transformer (GPT) 3.5 by OpenAI gained widespread attention in 2022 because it was made available as a chatbot in the “chatGPT” user interface and demonstrated impressive conversational skills. It was later succeeded by GPT-4, which displayed much-improved knowledge retrieval and logical reasoning capacities with far fewer hallucinations^7^.

A common application area under exploration for these LLMs is healthcare^7^. LLMs can be applied to medical problems through different approaches. One approach is to train these models specifically on medical data (Fig. 1A). One of the first language models to be successfully transferred to the medical domain was Bio-BERT, which showed robust capabilities for biomedical text mining^10^. Also, Google’s LLM “PALM” was fine-tuned on medical training data, resulting in Med-PaLM, which outperformed its previous version in medical use cases^11^. Recently, Google has introduced the next iteration, Med-PaLM 2, which scored a high 86.5% in the US Medical Licensing Exam (USMLE)^12^. Solving USMLE questions is a common benchmark test for LLMs, yet it is of limited use for real-world applications. However, fine-tuned LLMs have been shown to solve real-world problems such as predicting clinical outcomes just based on unstructured text data in EHRs^13^. Other ways to apply LLMs on medical problems do not require fine-tuning: Generalist LLMs can often be applied to medical tasks by just using a detailed input prompt. Another alternative is the use of “Retrieval Augmented Generation” (RAG) by which domain knowledge can be provided to a trained LLM in machine-readable format (Fig. 1B).

**Fig. 1:**
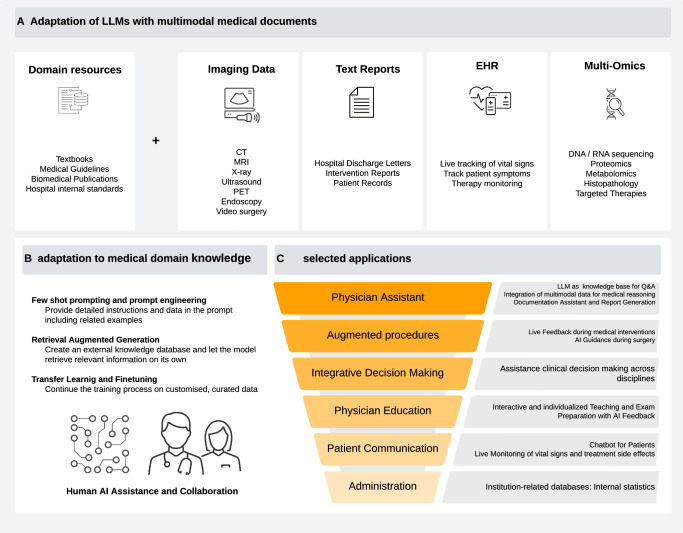
Overview of medical adaptations of LLMs. **A** Integrating information from various sources comprising domain knowledge from databases and patient related documents including different modalities (imaging, text, tabular, numerical data). **B** Possible model adaptation strategies include: Combining user prompts with relevant source documents and examples (few-shot prompting, top). Retrieval augmented generation (RAG), in which the information from A gets processed by a separate embedding model and is stored in a database. Relevant information can then be retrieved based on similarity measures with a user input (middle). Documents can be used to continue the training process and adapt the model parameters for specific use cases (bottom). Procedures are sorted by complexity and computational cost in increasing order. **C** Selected possible use cases for LLMs in clinical routine.

Today’s LLMs are transformer neural networks. This network architecture is well suited for almost any type of data, and enables multimodality. Multimodal AI systems are capable of interpreting multiple types of data together, such as textual and image data. Their development and validation require collaborative efforts between a number of disciplines including but not limited to medical experts in diagnostic specialties such as radiology or pathology and specialties such as surgery or medicine as well as technology experts both in software and hardware. Multimodal AI systems have been evaluated for various applications in precision oncology, such as outcome predictions^14,15^. However, more scientific evidence is required to ensure that LLMs and multimodal models provide quantifiable benefits in oncology.

Whenever a model is pre-trained on large and diverse tasks, and is subsequently applied to specialized tasks, it can be referred to as a “foundation model”^16^. Foundation models reduce the data requirements for specialized tasks, for example in predicting diseases from retinal photographs^17^. For instance, linking images from chest X-rays to corresponding report text data, and foundation models can alleviate the need for time-consuming and laborious manual annotation while preserving human-level accuracies and outperforming supervised methods^18^. In clinical practice, such models may be deployed in the form of chatbot assistants that can aid diagnosis in an interactive manner^19^. Similar examples exist in pathology, where large image datasets are linked with contextual knowledge and case-specific information yielding high performance in disease detection as well as biomarker prediction and can also inform further diagnostic procedures such as additional stains^20,21^. Furthermore, early generalist models have been introduced which show consistently high performance across a variety of medical domains and tasks integrating knowledge from diverse domains^22,23^. Given the resource-intensive nature of training models with parameter sizes in the billions, there is a trend towards improving model efficiency by reducing model size while retaining model performance. Recent advances with open-sourced models yield the perspective of de novo model training and development at much lower financial and computational burdens^24^.

As foundation models diversify their capabilities, they open up new avenues for their potential application in oncology and cancer research, such as multimodal diagnostics and drug discovery. However, to fully unlock the potential of foundation models in oncology and cancer research, several challenges must be addressed. Firstly, the underlying data the model is trained on has to be carefully assessed for quality, quantity, and diversity^25^. Secondly, the design of systems which integrate foundation models should be guided not only by experts in computer science, but also by medical professionals and patient advocates as well as the broader scientific community. Thirdly, the integration of such models in operable clinical software systems faces legal and regulatory challenges because these models require approval as medical devices^26^. Fourthly, ongoing model evaluation, validation and improvement are important to maintain quality, safety, and usefulness in light of the accelerating pace with which scientific discoveries are translated into novel medicines and guidelines. Lastly, a prominent concern of AI models based on neural network architecture is their often criticized lack of interpretability which earned them the term ‘black boxes’^27,28^. While substantial progress has been made in model explainability for image-related tasks, fewer studies address explainability in text processing or multimodal tasks in medicine^29^.

Overall, the advancements in LLMs and multimodal models have the potential to impact the practice of oncology through many different applications (Fig. 1C). This “Collection” in “npj Precision Oncology” aims to collect articles that provide solid empirical evidence for applications of these models in precision oncology.
